# Diagnostic imaging of the diabetic foot: an EANM evidence-based guidance

**DOI:** 10.1007/s00259-024-06693-y

**Published:** 2024-03-27

**Authors:** Chiara Lauri, Edel Noriega-Álvarez, Riddhika M. Chakravartty, Olivier Gheysens, Andor W. J. M. Glaudemans, Riemer H. J. A. Slart, Thomas C. Kwee, Frédéric Lecouvet, Emmanouil Panagiotidis, Jules Zhang-Yin, Jose Luis Lazaro Martinez, Benjamin A. Lipsky, Luigi Uccioli, Alberto Signore

**Affiliations:** 1 Inflammation and Infection Committee of the European Association of Nuclear Medicine (EANM), Vienna, Austria; 2 Nuclear Medicine Unit, Department of Medical-Surgical Sciences and of Translational Medicine, Faculty of Medicine and Psychology, “Sapienza” University of Rome, Rome, Italy; 3 Department of Nuclear Medicine and Molecular Imaging, University Hospital of Guadalajara, Guadalajara, Spain; 4 Radiology Department, Shrewsbury and Telford Hospitals NHS Trust, Shrewsbury, UK; 5 Department of Nuclear Medicine and Molecular imaging, University Hospitals Leuven, Leuven, Belgium; 6 Department of Nuclear Medicine and Molecular Imaging, University Medical Center Groningen, University of Groningen, Hanzeplein 1, 9700 RB Groningen, The Netherlands; 7 Department of Biomedical Photonic Imaging, Faculty of Science and Technology, University of Twente, Enschede, The Netherlands; 8 Department of Radiology, University Medical Center Groningen, University of Groningen, Groningen, The Netherlands; 9 Department of Radiology, Institut de Recherche Expérimentale et Clinique Cliniques, Universitaires Saint-Luc, Université Catholique de Louvain (UCLouvain), Brussels, Belgium; 10 Bone & Joint Committee of the European Association of Nuclear Medicine (EANM), Vienna, Austria; 11 Nuclear Medicine Department/PET CT, Theagenio Cancer Center, Thessaloniki, Greece; 12 Department of Nuclear Medicine, Clinique Sud Luxembourg, Vivalia, Arlon, Belgium; 13 Diabetic Foot Unit, Universidad Complutense de Madrid, Madrid, Spain; 14 Emeritus Professor of Medicine, University of Washington, Seattle, USA; 15 Green Templeton College, University of Oxford, Oxford, UK; 16 Diabetes and Endocrinology Section, CTO Hospital of Rome, Rome, Italy; 17 Department of Biomedicine and prevention, Tor Vergata University, Rome, Italy

**Keywords:** Infection diagnosis, Diabetic foot, Osteomyelitis, Imaging, Guidance

## Abstract

**Purpose:**

Consensus on the choice of the most accurate imaging strategy in diabetic foot infective and non-infective complications is still lacking. This document provides evidence-based recommendations, aiming at defining which imaging modality should be preferred in different clinical settings.

**Methods:**

This working group includes 8 nuclear medicine physicians appointed by the European Association of Nuclear Medicine (EANM), 3 radiologists and 3 clinicians (one diabetologist, one podiatrist and one infectious diseases specialist) selected for their expertise in diabetic foot. The latter members formulated some clinical questions that are not completely covered by current guidelines. These questions were converted into statements and addressed through a systematic analysis of available literature by using the PICO (Population/Problem–Intervention/Indicator–Comparator–Outcome) strategy. Each consensus statement was scored for level of evidence and for recommendation grade, according to the Oxford Centre for Evidence-Based Medicine (OCEBM) criteria.

**Results:**

Nine clinical questions were formulated by clinicians and used to provide 7 evidence-based recommendations: (1) A patient with a positive probe-to-bone test, positive plain X-rays and elevated ESR should be treated for presumptive osteomyelitis (OM). (2) Advanced imaging with MRI and WBC scintigraphy, or [^18^F]FDG PET/CT, should be considered when it is needed to better evaluate the location, extent or severity of the infection, in order to plan more tailored treatment. (3) In a patient with suspected OM, positive PTB test but negative plain X-rays, advanced imaging with MRI or WBC scintigraphy + SPECT/CT, or with [^18^F]FDG PET/CT, is needed to accurately assess the extent of the infection. (4) There are no evidence-based data to definitively prefer one imaging modality over the others for detecting OM or STI in fore- mid- and hind-foot. MRI is generally the first advanced imaging modality to be performed. In case of equivocal results, radiolabelled WBC imaging or [^18^F]FDG PET/CT should be used to detect OM or STI. (5) MRI is the method of choice for diagnosing or excluding Charcot neuro-osteoarthropathy; [^18^F]FDG PET/CT can be used as an alternative. (6) If assessing whether a patient with a Charcot foot has a superimposed infection, however, WBC scintigraphy may be more accurate than [^18^F]FDG PET/CT in differentiating OM from Charcot arthropathy. (7) Whenever possible, microbiological or histological assessment should be performed to confirm the diagnosis. (8) Consider appealing to an additional imaging modality in a patient with persisting clinical suspicion of infection, but negative imaging.

**Conclusion:**

These practical recommendations highlight, and should assist clinicians in understanding, the role of imaging in the diagnostic workup of diabetic foot complications.

**Supplementary Information:**

The online version contains supplementary material available at 10.1007/s00259-024-06693-y.

## Preamble

The European Association of Nuclear Medicine (EANM) is a professional non-profit medical association that facilitates communication worldwide amongst individuals pursuing clinical and research excellence in nuclear medicine. The EANM was founded in 1985. These guidelines are intended to assist practitioners in providing appropriate nuclear medicine care for patients. They are not inflexible rules or requirements of practice and are not intended, nor should they be used, to establish a legal standard of care. The ultimate judgement regarding the propriety of any specific procedure or course of action must be made by medical professionals, taking into account the unique circumstances of each case. Thus, there is no implication that an approach differing from the guidelines, standing alone, is below the standard of care. To the contrary, a conscientious practitioner may responsibly adopt a course of action different from that set out in the guidelines when, in their reasonable judgement of the practitioner, such course of action is indicated by the condition of the patient, limitations of available resources, or advances in knowledge or technology subsequent to publication of the guidelines. The practice of medicine involves not only the science, but also the art, of dealing with the prevention, diagnosis, alleviation, and treatment of disease. The variety and complexity of human conditions make it impossible to always reach the most appropriate diagnosis or to predict with certainty a particular response to treatment. Therefore, it should be recognized that adherence to these guidelines will not ensure an accurate diagnosis or a successful outcome. All that should be expected is that the practitioner will follow a reasonable course of action based on current knowledge, available resources, and the needs of the patient to deliver effective and safe medical care. The sole purpose of these guidelines is to assist practitioners in achieving this objective.

## Introduction

Diabetes-related foot disease is a global problem. Various types of foot lesions can occur in diabetic patients, usually as a consequence of the diabetes-related peripheral neuropathy, peripheral arterial disease, or both. These two factors lead to foot ulcers that may be caused by repetitive stress, development of foot deformities, micro-traumas, areas of high pedal pressure, excessive load and imbalance. Diabetes also frequently causes various, but mostly ill-defined, immunological perturbations. These, in the setting of a break in the protective skin envelope, often lead to the development of infection in the wound. This event, especially in presence of vascular impairment (which limits the migration of phagocytic cells and delivery of antibiotic therapy) dramatically complicates the management of foot complications.

The prevalence of active foot ulcers in persons with diabetes is about 6.3%, with rates of 5.5% in Asia, Europe and Africa, and 13% in North America [1]. Approximately a quarter of all persons with diabetes will, at some point during their lifetime, develop a foot ulcer [2]. These ulcers are the seeding point for the development of potentially serious infectious complications, starting with soft tissue infection (STI) and in many cases spreading to underlying bone, thus causing osteomyelitis (OM) [3].

The classical clinical symptoms used to define STI (with or without concomitant OM) are redness, warmth, swelling, pain and/or tenderness, and purulent secretions, usually in the setting of a foot wound or ulcer. The presence of various so-called “secondary” findings that should also raise suspicion of diabetic foot infection (DFI) include non-purulent secretions, friable and discoloured granulation tissue, wound undermining and foul odour [4]. Once infection of the diabetic foot is demonstrated, the severity is further classified as mild (involving only a limited area of the superficial skin and soft tissue), moderate (more wide spread infection, either horizontally or vertically), or severe (accompanied by systemic inflammatory response signs and symptoms) [5].

The spreading of the infection into the underlying bone is one of the most feared complications, as it is associated with high risk of lower extremity amputation, prolonged hospitalization, high social and financial costs and increased mortality rates [6–8]. Whilst the prevalence of OM underlying a non-healing diabetic foot ulcer is probably underestimated, its presence is clearly associated with worse outcomes [9], especially when radiographic changes are present [10].

Accurately diagnosing DFI is a clinical challenge, as the cardinal symptoms and signs of infection can be masked (or occasionally mimicked) by the presence of peripheral neuropathy or ischaemia, and positive wound culture results may only represent colonisation of the overlying soft tissues, rather than infection. As more than 50% of diabetic foot wounds are infected at their presentation [4], and the outcome of treatment is highly linked to how quickly the diagnosis of STI and OM are made, making an appropriate and prompt diagnosis is mandatory in order to avoid bad outcomes [11, 12].

A further issue complicating diagnosing diabetic foot lesions is the need to consider Charcot foot (CF), an inflammatory, potentially destructive foot disorder that mainly involves tarsal and metatarsal joints. If not promptly diagnosed and properly treated, it is a progressive degenerative musculoskeletal disease that can lead to crippling destruction of the foot [13]. The presence of neuro-osteo-arthropathy represents an additional diagnostic challenge for clinicians, since it may coexist with (or be the cause of) diabetic foot ulcers, and may lead to superimposed infections [14, 15]. Nevertheless, an accurate differential diagnosis between OM, STI and CF is crucial for the correct management, since these three conditions require very different treatments.

In 2008, the International Working Group on the Diabetic Foot (IWGDF) published a systematic review on treatment of diabetic foot OM, which only includes a “progress report” on diagnosis [16]. Clinical guidelines, from the Infectious Diseases Society of America (IDSA) and from the IWGDF have been published and updated [17–19], more recently in 2023 [5], provide diagnostic guidance, largely based on clinical aspects. The initial approach to a diabetic patient with a foot complication includes a detailed clinical history (especially of any recent but healed wounds, or antimicrobial therapy), a physical examination (especially for evidence of peripheral neuropathy or peripheral arterial disease of the affected foot), blood tests (for glycaemic control, routine chemistry and inflammatory markers) and plain X-rays of the foot. The findings of these evaluations, even in combination, can be inconclusive, thus requiring further advanced investigations [20].

The most commonly employed advanced imaging modalities are magnetic resonance imaging (MRI) and various types of nuclear medicine (NM) examinations, including radiolabelled white blood cells (WBC) scintigraphy and fluorine-18 Fluorodeoxyglucose positron emission tomography/computed tomography ([^18^F]FDG PET/CT) [20–22]. Whilst these studies are more expensive and may be less available than X-rays, they are more sensitive and specific, thus often necessary to accurately assess for the presence of foot complications.

From a NM point of view, several guidelines and consensus documents have been published by the EANM, including on bone scintigraphy [23], WBCs labelling procedures [24, 25], acquisition protocols and interpretation criteria [26] and on imaging infections and inflammations with [^18^F]FDG PET/CT [27]. Similarly, the acquisition protocols of several radiological imaging modalities in musculoskeletal disorders are now well standardized [28–30]. As this consolidated background information about DFI should represent the starting point of medical decision-making and actions, imaging specialists should always refer to the existing guidelines in their daily practice.

Moreover, several reviews, systematic reviews and meta-analyses have been published exploring the potential of NM techniques in detecting DFO [7, 31–36], reaching different conclusions. From these studies, it emerges that there is a wide heterogeneity in patient populations and diagnostic approaches, thus causing a large variability in reported accuracies for different imaging techniques.

Overall, there is no longer a role for the three-phase bone scan, except in excluding an infection (because of its high negative predictive value). In cases of equivocal MRI findings, performing WBC imaging or an [^18^F]FDG PET/CT is appropriate, but for the latter, well-standardized interpretation criteria are still needed.

Nevertheless, despite the publication of many systematic reviews and meta-analyses and of the American guidelines on imaging diabetic foot OM [29, 37], there is still no widely accepted evidence-based guidance for clinicians and imaging specialists in selecting the most appropriate diagnostic tests for detecting other foot complications, such as STI and complicated CF.

## Purpose of this document

These practical evidence-based recommendations on imaging of diabetic foot complications are designed to assist diabetologists, orthopaedic surgeons, radiologists, nuclear medicine physicians and other specialists dealing with these patients, to identify the most appropriate and tailored diagnostic strategy in cases of suspected DFI.

To this purpose, we herewith provide evidence-based recommendations based on practical clinical questions, for achieving effective and safe medical care.

## Methods

### Working group and strategy

The Inflammation & Infection Committee of EANM created a working group, including radiologists, diabetologists, podiatrists, and infectiologists, with expertise in DFI. The clinicians formulated important clinical questions that should be addressed by imaging specialists. These were the starting point for defining several statements that were then used to perform a literature search based on the PICO (Population/Problem–Intervention/Indicator–Comparator–Outcome) strategy. Papers of interest were graded by their level of evidence and used to formulate final recommendations. These were then graded according to the Oxford Centre for Evidence-Based Medicine (OCEBM) criteria [38, 39]. Selected papers for each statement were analysed and scored by all members of the writing group, and after several revisions, all delegates approved the final version of this document.

### Statements

Uniform statements were developed with the aim of providing evidence-based answers to the formulated clinical questions. These were designed to take the most relevant issues into consideration, including the: availability of the diagnostic procedures; patient acceptance and tolerability; risk of complications; and, financial costs. Each consensus statement is followed by comments based on an analysis of the available literature, and by a conclusive recommendation. This approach was designed to provide practical information that would be relevant, in daily practice, for patient management.

### Literature search

Following the recommendations of the Oxford Centre for Evidence-Based Medicine and of the Cochrane system, the writing group performed a literature search, from January 2000 to May 2023, using the PubMed/Medline and Scopus databases. A cross-search based on references included in the retrieved articles, along with a hand search of other papers known to the authors were also performed to seek any additional articles. Search terms were defined by agreement with all members of the writing group. Inclusion of papers supporting each statement was based on a PICO question that was converted into a search strategy, as described by OCEBM [38, 39]. Case reports, abstracts, papers with less than 10 patients and those not published in English language were excluded. Systematic reviews, however, were included.

Search results for each statement are summarized in the Appendix (see Supplementary Information).

### Scoring system and recommendation grading criteria

All included papers used to address each statement were carefully read and analysed by the members of the writing group. A “level of evidence” for each paper was assigned in consensus with all delegates, according to the procedure described in the OCEBM [38, 39]. At the end of each statement, a final recommendation was also provided and graded, again in agreement with all delegates, based on the average of paper scores.

## Clinical questions and practical recommendations

### Question 1

For patients with suspected foot OM, the IWGDF guidelines recommend using a combination of the probe-to-bone (PTB) test, erythrocyte sedimentation rate (or C-reactive protein and/or procalcitonin), and plain X-rays as initial diagnostic steps [19]. However, not all of the diagnostic findings will be present in each case of OM, since all of them have a relatively low sensitivity and specificity. It is, therefore, necessary, in some cases, to consider supplementing the diagnostic process with an advanced imaging modality.

- *1a*: Is it true that patients with some combination of a positive PTB, changes in plain X-rays, local inflammatory signs, or elevated serological inflammatory markers (Erythrocyte sedimentation rate, C-reactive protein, and procalcitonin) have OM?
- *1b*: Do imaging modalities add any relevant information in patients with a combination of these clinical and laboratory findings used to diagnose OM?
- *1c*: What imaging modality should be performed in patients with positive results on one or more of these clinical and laboratory findings?

### Reply to question 1

To address the value of these various clinical and laboratory tests, we performed a single broad search, retrieving 23 papers (see Appendix 1 in Supplementary Information). After review, 14 papers were retrieved and 3 additional papers, selected from references, met our criteria and were included.

*1a*: Inflammatory markers are useful to alert the clinician to the possibility of an infection, but they are not sufficiently specific and not able to define the severity of the process. Although different thresholds have been proposed, mainly for ESR [40–43], they showed fair accuracy in detecting OM, especially if used alone [36, 42].

The accuracy of PTB test largely depends on doing the test correctly and the clinician’s experience. Available studies show a moderate inter-observer agreement. The accuracy of the tests depends on the ulcer’s anatomic location, its aetiology (ischemic, neuro-ischemic or neuropathic) and, most importantly, on the pre-test probability of the studied populations, being high in high-risk patients but only fair in patients with low pre-test probability [44]. Therefore, a positive test is useful, but a negative test requires additional diagnostic studies [32, 45, 46].

It is commonly accepted that plain X-rays, being widely available and relatively inexpensive, should be the first line imaging modality to perform. However, bone abnormalities can be caused by non-infectious processes and sufficient bone loss to be easily detectable takes approximately two weeks. This makes X-rays results alone, only marginally useful if positive and even less useful when negative. Moreover, interpreting them without the knowledge of the patient’s clinical history and physical assessment and laboratory results may result in a misdiagnosis [47].

Available data suggest that a combination of the clinical, laboratory and radiological tests is useful for an initial screening of the patients with suspected OM [4, 16, 19, 36, 41]. In particular, the presence of an ulcer area >2 cm^2^, a positive PTB test and an ESR >70 mm/h increase the likelihood of OM, especially if associated with abnormal X-rays [41].

One large prospective study of 338 patients found that the simple sequential approach of performing a PTB test and plain X-rays was quite accurate for diagnosing an OM, especially when both tests are positive, with a sensitivity of 97% and a specificity of 93% [48].

Based on these studies, most of them using histology as reference, systematic reviews and meta-analyses, and clinical guidelines, we can answer the first clinical question. The presence of a combination of a positive PTB test, substantially raised inflammatory markers and suggestive X-rays makes the presence of OM highly likely [47].

*1b*: The appeal to advanced imaging with MRI and hybrid imaging with single-photon emission computed tomography (SPECT) or PET is important in providing clinicians helpful information about the extent of bone infection, its precise location, and any involvement of surrounding soft tissues, in therapy decision-making [14, 15, 20, 21, 49, 50]. This information cannot be completely and accurately evaluated by using only clinical, laboratory or plain X-ray results.

Meyr et al. calculated the level of agreement between PTB test, X-rays, MRI, histology and microbiology, reporting low levels of inter-test agreement between several commonly used diagnostic tests (range 42 to 62%). Although fair, the highest level of agreement was between X-rays and MRI. These results underline the need of a multimodal approach, however the specific combination of tests that should be used remains unclear [49]. MRI allows the detection of abnormalities not detected by these other tests, such as abscesses, tenosynovitis, and joint involvement, thus providing more accurate information about the extent of involvement of bone and soft tissue [50]. Nevertheless, MRI may fail in differentiating OM from Charcot neuroarthropathy, especially when primary and secondary signs are subtle and the bony architecture is extensively compromised. Moreover, determining whether a CF has a superimposed infection can be a challenge with MRI. NM, especially WBC imaging including SPECT/CT, may be particularly helpful in these situations [15].

*1c*: We found only one large prospective study that addresses the issue in this question. In 2014, Zaiton et al. studied 102 patients with clinical suspicion of diabetic foot OM based upon having an infected foot ulcer, but who had negative X-rays. All of the patients had a PTB test and MRI examination [50]. The presence or absence of OM was determined by the results of bone histology, which was positive for 78% of the patients. For MRI, the sensitivity was 98% and specificity 89%. For the PTB test, the sensitivity was 83% and specificity 77%. Thus, in this series of patients with clinical suspicion of OM despite negative X-rays, PTB was useful, but considerably less accurate than MRI [50]. Unfortunately, we found no similar well-designed studies on other imaging techniques, therefore, it is not possible to definitively assess which is the best modality for patients like those in this study.

In examining the published meta-analyses comparing different techniques for diagnosing OM, we found conflicting results. A 2008 meta-analysis by Dinh et al. included 9 studies and found that MRI emerged as the most accurate test for diagnosis of OM, with a pooled diagnostic odds ratio of 24, considerably higher than plain X-rays, bone scan or WBC imaging [32]. Exposed bone or PTB had a high pooled diagnostic odds ratio, but this was based on only two studies. WBCs were radiolabelled with ^111^In and performed with dated acquisition protocols, mainly without SPECT/CT, and interpretation criteria thus, possibly underestimating the accuracy of this modality [32]. Conversely, in the 2006 meta-analysis by Capriotti et al., ^99m^Tc-WBC scan showed higher specificity and accuracy compared to MRI (respectively, 84.5% and 86% vs 74% and 80.5%) [7]. Similar conclusions emerged from a more recent systematic review and meta-analysis by Lauri et al., where the specificity for ^99m^Tc-WBC and [^18^F]FDG PET/CT (92% for both) was higher than MRI and ^111^In-WBC (both 75%), whereas all the techniques showed a similar sensitivity (approximately 93%) [31].

Thus, based on the available meta-analyses, we are not able to offer a firm answer to this specific clinical question.

Of note, both IWGDF/IDSA guidelines [5] and the Society for Vascular Surgery in collaboration with the American Podiatric Medical association and the Society for Vascular Medicine [45] suggest using MRI for those patients who require a more sensitive or specific imaging study, or when the diagnosis of OM is uncertain with less advanced tests. If MRI is contraindicated or not available, WBC imaging, or in alternative [^18^F]FDG PET/CT, should be performed.

More prospective well-designed studies are warranted to define which is the best technique or the best combination able to accurately diagnose an OM.

**Table:** 

***Recommendations***	***Grade***
• A combination of a positive PTB test, an elevated ESR and a positive X-ray makes the presence of OM highly probable and no additional imaging modalities are required for diagnosis.	*B*
• Additional imaging with MRI may provide useful additional information on the extent of both bone and soft tissue infection.	*B*
• If, however, PTB is the only positive test, advanced imaging modalities (MRI or WBC imaging or [^18^F]FDG PET/CT) should be considered, especially in high-risk patients.	*B*

### Question 2

IWGDF guidelines recommend performing an advanced imaging study, such as MRI, or [^18^F]FDG PET/CT or WBC imaging, only in cases in which the diagnosis of OM remains doubtful after routine studies.

- *2a*: Which imaging modality is more accurate in diabetic patients with suspected OM of the fore-mid-foot?
- *2b*: Which modality is more accurate in cases of OM in the mid- or hind-foot?

### Reply to question 2

*2a*: Our review included 16 studies (8 retrospective studies, and 8 reviews) but only one of them compared, separately, the value of different imaging modalities in the fore- and/or mid-foot [15].

The multicentre retrospective study performed by Lauri et al. on 251 patients compared WBC imaging, [^18^F]FDG PET/CT and MRI in patients with OM, STI and CF, either in any site and according to the location. Compared to mid-hindfoot OM, WBC scintigraphy showed significantly higher sensitivity and [^18^F]FDG PET/CT showed significantly higher sensitivity and accuracy in detecting forefoot OM. MRI did not show a significantly different performance in fore or mid-hindfoot OM. Overall, none of the three imaging modalities showed a significant superiority according to different location [15].

The other included studies only analysed the distribution of pedal OM arising from different aetiologies, including diabetes, based on MRI findings. Three studies were from the same research group in the same year in almost the same patient groups [51–53]. Overall, the most frequent location of OM was in the forefoot, specifically involving the fifth metatarsal, first metatarsal, and first distal phalanx, all directly adjacent to skin ulcers or surgical defects [51]. Despite MRI emerges as the imaging modality of choice to detect abscesses in patients with pedal OM [52] and to detect necrotic tissue, by its lack of gadolinium enhancement, the presence of areas of devascularisation may also be characterized by lack of enhancement. This pitfall must be taken into consideration since it may mask the presence of abscess and OM [53, 54]. In those cases, NM techniques might be an option. By detecting or excluding underlying OM in diabetic patients with a neuropathic forefoot ulcer, MRI may also be helpful in guiding therapy decision-making, thus simplifying the choice between conservative management [55] and surgical approach [56].

One study from 2003, compared ^99m^Tc-labelled monoclonal anti-granulocyte antibodies for diagnosing pedal OM to ^111^In-WBCs in 25 diabetic patients with pedal ulcers (22 in the forefoot, 3 in the mid-foot). The operating characteristics for the two techniques for diagnosing pedal OM were comparable, with a sensitivity of 80 to 90% and specificity from 72 to 76%, respectively [57].

As previously mentioned, several reviews have described the value of imaging modalities for assessing various diabetic foot diseases [7, 31–36, 58]. One, published in 2003, before the era of hybrid camera systems, found that for diagnosing OM (with no distinction between fore-, mid-, or hind-foot): radiography had a sensitivity of 28–93% and specificity 25–92%; three-phase bone scan had a sensitivity of 67–100% and specificity 18–83%; and WBC imaging had a sensitivity of 75–100% and specificity 54–89%. The use of [^18^F]FDG PET/CT scanning was not included in this review [58]. The other descriptive review, published in 2009 [34], stated that WBC imaging is the NM procedure of choice for investigation of diabetic foot infections, with an overall accuracy of 80–85%. SPECT/CT will likely improve diagnostic accuracy even further, especially in the mid- and hind-foot. Bone scans were found to be of questionable value, and data on [^18^F]FDG PET/CT were limited and inconclusive [34]. Despite more recently published meta-analyses on [^18^F]FDG PET/CT [31, 33, 35] report wide range of accuracies, mainly depending on the type and number of the included studies, this modality emerges as useful and non-invasive tool for detecting DFO. Nevertheless, the development of standardized interpretation criteria is still needed to further improve its accuracy and for the harmonization of this diagnostic approach.

Overall, as it also emerges from a recent meta-analysis published by Llewellyn et al., MRI, WBCs and [^18^F]FDG PET/CT have a similar high accuracy and there are no evidence-based data to definitively prefer one over the other imaging modalities [35]. Based on available data, there is no longer a role for the three-phase bone scan, except in excluding an infection. In MRI cases with equivocal findings, either WBC imaging or [^18^F]FDG PET/CT could be selected, depending on availability and local preferences.

*2b*: In total, 11 studies were included (8 retrospective studies and 3 prospective study) that at least partly evaluated the value of imaging modalities in the mid- and/or hind-foot. Some of these performed a sub-differentiation regarding the anatomic regional location of the OM.

Three retrospective studies have evaluated the role of MRI [51, 59, 60], three retrospective and one prospective study evaluated the WBC scan [57, 61–63], two prospective studies compared MRI and [^18^F]FDG PET/CT [64, 65] and two combined retrospective studies performed [^18^F]FDG PET/CT, WBC scan and MRI, and WBC scan and MRI, respectively [15, 66]. The sensitivity for detection of OM in the mid- or hind- foot ranges from 40 to 100% for MRI, with a specificity ranging from 61% to 82% [15, 51, 66].

In a retrospective study specifically analysing different foot regions, [^18^F]FDG PET/CT provided a sensitivity of 53% and a specificity of 73%; WBC scan was 71% sensitive and 85.7% specific and MRI resulted in 77% of specificity and only 40% of sensitivity in detecting mid-/hind- foot OM. Nevertheless, no significant differences between the three modalities according to different locations were observed [15]. In general, studies of WBC scan on diagnosing OM in the diabetic foot reported a sensitivity in the mid-/hind-foot ranging from 71–93%, with a specificity of 71–99% [15, 61, 62, 66].

In a prospective study on 31 patients, performed by Garcia-Diez et al., the authors compared diffusion-weighted imaging (DWI) and dynamic contrast-enhanced (DCE)-MRI and [^18^F]FDG PET/CT in differentiating OM from uncomplicated CF [64]. Although several MRI parameters allowed a reliable distinction, mainly when large ROIs were used, visual assessment of [^18^F]FDG biodistribution performed better than MRI with a sensitivity, specificity, positive predictive value (PPV) and negative predictive value (NPV) of 88.9%, 96%, 86.4% and 95.9%, respectively. Semi-quantitative parameters did not provided significant improvement compared to visual assessment [64].

Previously, Basu et al. investigated the added role of [^18^F]FDG PET, without CT co-registration, over MRI in differentiating OM from CF in mid-hindfoot disorders [65]. [^18^F]FDG PET showed the highest NPV in ruling out OM in patients with concomitant Charcot. CF without a superimposed OM showed mild and diffuse uptake with a SUV_max_ ranging from 0.7 to 2.4. In the only patient with concomitant OM the SUV_max_ was significantly higher (6.5). Despite both modalities correctly diagnosed the OM in this patient, MRI showed high number of FP results in uncomplicated CF and FN results in patients with proven STI, that were correctly identified by [^18^F]FDG PET. The authors, therefore, concluded that [^18^F]FDG PET allows a reliable differentiation between uncomplicated CF and CF with a superimposed OM [65]. Nevertheless, these findings, and in particular the reliability of interpretation criteria for [^18^F]FDG PET in these challenging clinical scenario, should be confirmed by further solid studies.

Published reviews comparing the accuracy of using MRI, WBC scan and [^18^F]FDG PET/CT in diagnosing OM in the mid- and hind-foot in diabetic persons are lacking. Lauri et al. reported on the value of these three imaging techniques in diagnosing diabetic foot OM [31]. The performance characteristics were as follows: for [^18^F]FDG PET/CT sensitivity 89%, specificity, 92%; for WBC scan with ^111^In-oxine and ^99m^Tc-HMPAO, sensitivity 92% and 91%, respectively, and specificity 75% and 92%, respectively; for MRI, sensitivity 93%, specificity 75%. Although this systematic review and meta-analysis did not examine the diagnostic performance of the three imaging modalities according to the different foot location, both WBC scan and [^18^F]FDG PET/CT showed an overall high accuracy in detecting OM in any site.

Concluding, MRI is useful for detecting soft tissue abscesses and necrotic tissue, but might miss OM in these areas. Based on the available data, in cases with equivocal results on MRI, clinicians can select either WBC or PET/CT imaging. In very complicated cases, e.g., in post-traumatic or post-operative phases, in which both MRI and [^18^F]FDG PET/CT may fail due to their limited specificity in differentiating infection from sterile inflammation, or in the presence of lower limb ischemia, WBC SPECT/CT imaging may be useful.

In summary, there are no evidence-based data to definitively determine the most accurate imaging modality to use in diabetic patients with suspected OM of the mid- and hind- foot and the choice should be based on the single clinical case.

**Table:** 

***Recommendation***	***Grade***
In case of equivocal results on MRI, radiolabelled WBC imaging or, in alternative, [^18^F]FDG PET/CT should be used to detect OM.	*C*

### Question 3

IWGDF guidelines recommend to diagnose a STI in diabetic foot clinically, based on the presence of local or systemic inflammatory signs and symptoms, and to classify the severity of the infection according to the ulcer depth, the extent of spread of cellulitis from the ulcer, and the presence of systemic signs (inflammatory response) [5]. Whilst mild infections may be treated with oral (and perhaps topical) antimicrobial therapy, some moderate and all severe infections require urgent interventions, generally including intravenous antibiotic therapy, surgical debridement and patient stabilization. The decision to perform a surgical debridement is based on the clinical presentation, but sometimes it is not possible to estimate either the severity of the infection or the extent of tissue involvement. Therefore, we need to know the following:

- ***3a*:** What kind of imaging modality is recommended in patients with an ulcer located at fore-/mid-foot complicated by a severe or moderate infection before performing a surgical debridement of the infected tissue?
- ***3b*:** Which imaging modality is recommended if the infected ulcer is located in the hind-foot?

### Reply to question 3

***3a*:** Early diagnosis and appropriate treatment can lead to successful healing of diabetic foot ulcers, reducing the need for surgery. Although the diagnosis of STI is usually achievable by clinical examination, imaging may be sometimes useful to assess the extent of the process. Nevertheless, some imaging modalities may fail in differentiating between sterile inflammation and infection.

Plain X-rays are not optimal for detecting soft tissue abnormalities, but a recent retrospective study on 62 diabetic patients reported that the most frequent primary location of soft tissue emphysema, detectable with X-rays, was the forefoot (61.3%), followed by the mid-foot (21.0%) and hind-foot (16.1%). The soft tissue emphysema was most frequently observed in the dorsal foot tissue (49.2%), followed by both dorsal and plantar tissue (27.4%), and the plantar foot tissue (24.2%). X-rays findings showed a good correlation with CT findings, treatment outcome and microbiology [67].

Using CT to evaluate forefoot structures in 32 patients, Robertson et al. reported significant differences between age-matched controls and individuals with diabetes and a previous plantar ulcer. They noted that plantar muscle density was decreased, and metatarsophalangeal joint extension and arthropathy were increased in diabetic patients. Interestingly, the soft-tissue thickness under the metatarsal heads did not differ between the two groups. They concluded that CT may guide the need for further interventions for prevention or treatment of foot ulcers in individuals with diabetes [68].

One non-randomized controlled trial by Commean et al. used 3D spiral X-ray computed tomography imaging methods to measure anatomic foot structure in diabetic patients with a forefoot ulcer. They found these methods useful for determining structural differences between diabetic patients and those with “healthy” feet and for evaluating how these differences relate to plantar pressures, thus providing information for planning treatment [69].

MRI with fluid-sensitive, fat-suppressed sequences is generally considered the modality of choice for investigating soft-tissue complications, as it helps defining the extent of the soft tissue process with higher tissue contrast ratio than X-rays or CT. MRI can also identify underlying skin ulcers, sinus tracts, abscesses, and tenosynovitis, as well as differentiating cellulitis (showing enhancement after intravenous contrast administration) from simple edema (no enhancement).

Pedal OM results almost exclusively from contiguous spread of infection from soft tissue to bone, and Ledermann et al., in 2002, reported that it occurs most frequently around the fifth and first metatarsophalangeal joints [51]. The same group reviewed contrast-enhanced MRIs of the foot in persons who had a bone biopsy or surgery for suspected OM. They found that soft-tissue inflammation in the forefoot is not always confined in fascial planes but can spread into adjacent compartments, whereas hind-foot infection tends to be confined and only rarely spreads to other compartments [70].

WBC imaging with SPECT/CT acquisitions is considered the standard procedure for investigating patients with suspected STI. Petruzzi et al. reported sensitivity and specificity values ranging from 86 to 90%, with a slightly higher sensitivity for acute processes [71]. Heiba et al. used a combination of ^111^In-labelled WBC + SPECT/CT scintigraphy and bone scan to evaluate patients with a DFI, concluding that dual isotope SPECT/CT was superior than bone scan or WBC SPECT/CT alone in discriminating STI from OM [61]. In another study of 272 patients with suspected diabetic foot infection, they concluded that this combined approach is associated with a reduced length of hospitalization [72].

Although [^18^F]FDG PET/CT is not recommended for differentiating between septic and aseptic inflammation, it has been used for assessing ulcers and OM in diabetic patients. Keidar et al. reported that the detection of an [^18^F]FDG avid focus located to the bone or to soft tissue, allowed planning the most appropriate treatment [73]. Similarly, Familiari et al. found that using a dual-acquisition [^18^F]FDG PET/CT protocol could be helpful for differentiating between OM and STI, with CT component of primary relevance [74].

A multicentre retrospective study, WBC scan showed higher, although not statistically significant, sensitivity than [^18^F]FDG PET/CT and MRI (75% vs 27.3% vs 42.9%, respectively) in detecting STI but both PET/CT and WBC scan were significantly more specific than MRI (97.9% and 95.7% vs. 83.6%, *p*=0.04 and *p*=0.018, respectively). Nevertheless by analysing their performance according to the different locations, the modalities were comparable [15].

Concluding, clinicians can select MRI, WBC SPECT/CT or [^18^F]FDG PET/CT to plan the treatment of fore- or mid-foot ulcers. MRI is generally performed before NM imaging since it is widely available and it is a radiation-free imaging technique. If findings on MRI are compatible with OM, the patient should be treated for bone infection; if not, such treatment is unnecessary. WBC SPECT/CT or [^18^F]FDG PET/CT could be helpful when clinical signs and symptoms and radiologic findings are incongruent, when MRI cannot be performed for technical reasons or in patients with MRI equivocal results. If either WBC SPECT/CT or [^18^F]FDG PET/CT suggest the presence of OM, the patient can be properly treated for this disease; if the study is most compatible with STI, OM treatment may be withheld, but patients should be followed-up to assess for healing or development of OM.

***3b*:** To try to answer this clinical question, we retrieved 21 papers but only included 5 studies (4 retrospective and one prospective) [15, 51, 61, 70, 75].

Changes on X-rays are not sensitive, as they require several weeks to allow the visualization of sufficient demineralization. Furthermore, changes are also frequently nonspecific.

MRI, as demonstrated in one study from 2002 of patients with pedal OM (most of whom had diabetes), had an overall high sensitivity (90%) and acceptable specificity (79%) for detecting OM, but its diagnostic performance in STI significantly varies across different regions of the foot [51]. Ledermann et al. in a study of 115 patients with suspected pedal OM, amongst whom the primary infected compartment in 21 was the hind-foot, reported that the process tends to remain confined (heel ulcers being the most frequent focus of infection), and only 7% of cases showed spread from the hind-foot and malleoli to adjacent compartments [70].

Heiba et al., by performing sequential bone scan and WBC imaging, and if needed, bone marrow scintigraphy (BMS) with SPECT/CT, concluded that dual isotope SPECT/CT was helpful in achieving a correct diagnosis in patients with mid-/hind-foot infection and was highly accurate in discriminating STI from OM [61]. Moreover, it is known that in the mid-/hind-foot, the specificity of WBC imaging may be hampered by bone expansion associated with fractures and, in particular, with a neuropathic joint [14, 61, 74, 76]. The addition of a BMS could therefore be crucial, particularly when assessing the mid-/hind- foot, to correctly discriminate between OM from Charcot [61, 76]. Therefore, this combined imaging technique could improve the diagnostic accuracy of foot pathology in diabetic patients.

A study by Keidar et al. evaluated [^18^F]FDG PET/CT uptake for detecting pedal OM in patients with suspected pedal infection [73]. In 14 such patients, they found that PET detected 14 foci of increased [^18^F]FDG uptake, suspected as infection in 10 patients. PET/CT correctly localized 8 foci in 4 patients to bone, and correctly excluded OM in 5 foci in 5 patients. They found PET/CT localized infection to bone or soft tissue with an accuracy of 94% [73]. On the other hand, another prospective study on 20 diabetic patients with persistent pedal ulcers, MRI was superior to [^18^F]FDG PET in detecting foot ulcer–associated OM (90% versus 70%, respectively) [75]. Nevertheless, it is worthwhile to mention that these results were achieved by using stand-alone PET without PET co-registration, thus possibly underestimating the diagnostic performance of PET [75].

The use of hybrid imaging, with both SPECT/CT and PET/CT, radically improves the diagnosis allowing an accurate definition of anatomic landmarks of infective foci and thus discriminating superficial from deep infections.

As previously mentioned, Lauri et al. recently compared WBC imaging, [^18^F]FDG PET/CT, and MRI according to the location of STI. [^18^F]FDG PET/CT was found to be more specific than MRI in detecting STI in the mid-/hind- foot (*p*=0.03) with a comparable sensitivity. This study, therefore, suggests that both nuclear medicine modalities may accurately achieve a correct diagnosis. It also confirmed the higher accuracy of WBC scintigraphy, particularly when EANM guidelines were adhered to and completed with SPECT/CT, in discriminating pedal OM, STI, and Charcot arthropathy regardless their location [15].

Overall, as stated before for OM, a clear superiority of one of these three imaging modalities in detecting STI in different foot locations did not emerge, to reach definite conclusions.

**Table:** 

***Recommendation***	***Grade***
MRI, WBC scan and [^18^F]FDG PET/CT have a comparable accuracy in detecting infected ulcers in any site. In case of equivocal MRI findings, hybrid imaging with both WBC scan and [^18^F]FDG PET/CT can be used.	*C*

### Question 4

Foot ulcers may complicate a CF, in both its acute and chronic stages and OM may develop. What imaging modality should be performed when there is suspicion of OM complicating a CF?

### Reply to question 4

Given the progressive, destructive nature of Charcot’s arthropathy, a delay in the diagnosis can result in the progression of deformity, increasing the risk of ulceration, often followed by superimposed infection, potentially leading to amputation. The most common anatomic site affected by Charcot is the mid-foot and, in particular, the metatarso-cuneiform and naviculo-cuneiform joints (type I arthropathy) [77].

Because there is no definitive single method to consistently differentiate Charcot arthropathy from OM, a combination of physical examination, laboratory tests, and imaging has to be used to guide diagnosis, and thereby treatment [78].

The accuracy of X-rays in differentiating OM from Charcot arthropathy is approximately 50 to 60%, when demineralization, periosteal reaction, cortical destruction and/or periosteal new bone formation are present [77, 79]. However, these findings may not appear until 2 to 3 weeks after the onset of clinical symptoms, as they require the loss of 40% to 50% of bone mass to be detectable [11, 79, 80]. Although recently Mens et al. proposed the use of dual-energy CT (DECT) to quantify the presence of bone marrow edema in patients with ulcers and suspected OM, as alternative to MRI [81], there is no relevant literature to support the use of CT in these patients, unless surgical reconstruction is planned [11].

As previously mentioned, MRI is the imaging modality of choice for investigating the possibility of OM in the feet, having both high sensitivity (77–100%) and specificity (80–100%) [77, 82–84]. Unsurprisingly, distribution of OM mirrors that of ulceration, which is most common at the toes, metatarsal heads, calcaneus, and malleoli. On the contrary, neuropathic arthropathy is most common in the midfoot, particularly at the Lisfranc and Chopart joints [78, 83]. Of note, MRI often fails to distinguish bone marrow edema associated with Charcot arthropathy from that caused by OM. In the acute and subacute phases of Charcot’s neuroarthropathy bone marrow edema is characterized by increased bone marrow signal on T2 and STIR sequences, thus mimicking OM [65, 85].

When interpreting an MRI examination, the clinician and radiologist must consider the pattern of bone marrow edema, presence of ulceration and clinical correlations. As > 90% of OM on the foot/ankle result from contiguous spread of infection that starts in the skin, most cases have some combination of “secondary signs”, such as adjacent skin ulceration, cellulitis, soft tissue abscess or sinus tract [70, 83].

NM may improve the accuracy of diagnosis of OM. Bone scans are hampered by a low specificity, resulting from a high-rate false positive results related to bone remodelling, trauma, arthritis, recent surgery, or Charcot arthropathy with or without superimposed infection [78].

Compared to MRI, WBC imaging may be more effective in differentiating OM from Charcot arthropathy, and it can be used in patients with metal implants. However, false positive results may be observed in patients with increased bone marrow expansion, commonly observed in non-infected Charcot arthropathy, which may mask the presence of a superimposed OM [14, 86, 87].

The combination of WBC imaging and bone marrow scan may increase diagnostic accuracy [86, 88]. SPECT/CT is also largely used to improve the detection and localization of CF with superimposed OM or STI [61].

PET/CT may be used as an alternative to MRI or WBC imaging, but due to the lack of well-standardized criteria, interpretation of the findings often relies on the evaluation of uptake intensity and its location [65, 86, 89].

Basu et al., in a prospective comparative study on 63 patients, reported higher sensitivity and accuracy for [^18^F]FDG PET/CT compared to MRI in differentiating uncomplicated Charcot’s neuroarthropathy from OM and STI (for [^18^F]FDG: 100% and 93.8%, respectively; for MRI: 76.9% and 75%, respectively) [65]. Moreover, they observed a low degree of diffuse [^18^F]FDG uptake in the Charcot’s joints, with a SUV_max_ ranging from 0.7 to 2.4, that was clearly distinguishable from the normal joints and the uncomplicated diabetic foot (from 0.2 to 0.7 and from 0.2 to 0.8, respectively). Conversely, Garcia-Diez et al. did not find any added contribution of semi-quantitative analysis over qualitative assessment of [^18^F]FDG biodistribution and concluded that visual analysis is more accurate than MRI in differentiating OM from uncomplicated Charcot [64].

Hopfner et al., in two studies on the use of PET in Charcot’s osteo-arthropathy, using surgery as the criterion standard, reported a higher detection rate for PET compared to MRI [90, 91]. Rastogi et al. aimed to assess the efficacy of [^18^F]-fluoride PET/CT and [^18^F]FDG-labelled autologous leukocytes ([^18^F]FDG-LL PET/CT) in comparison with contrast enhanced MRI for the detection of diabetic foot OM complicating CF. They reported a sensitivity and specificity of 83.3% and 100% for [^18^F]FDG-LL PET/CT and 83.3% and 63.6% for MRI, respectively [89].

Concluding, in case of an erythematous and/or edematous foot without an ulcer or characteristic radiographic changes, MRI is useful to rule in or rule out CF. In the presence of an open wound, MRI appears to be of limited help. In these cases, WBC imaging is more sensitive and specific for OM, even in presence of concomitant Charcot arthropathy. PET/CT may also be helpful, but well-standardized interpretation criteria are lacking [88, 92].

**Table:** 

***Recommendation***	***Grade***
MRI has several limitations in diagnosing CF. WBC imaging is sensitive and specific for OM, even in presence of concomitant Charcot’s arthropathy. As an alternative, [^18^F]FDG PET/CT may be used	*B*

### Question 5

Once a diagnosis of OM of the diabetic foot is made, most published articles recommend treating patients with antibiotics for 6 weeks. Some data suggest that this may be reduced (perhaps to 3 weeks, or even less) in some specific conditions, such as when all necrotic and most infected tissue has been surgically removed. But it is still not clear how to determine if OM has been cured (or, perhaps more accurately, put into remission) at the end of treatment. What kind of examination should be performed to confirm that OM is cured after 6 weeks of antibiotic treatment?

### Reply to question 5

Promptly evaluating the effectiveness of medical treatment of OM is important, as it helps clinicians know when they can discontinue antibiotic therapy, and whether they need some surgical intervention. Shortening antibiotic therapy duration can reduce the risk of drug-related side effects, antibiotic-resistance, and unnecessary financial costs. Currently, there is no single clinical finding, laboratory test, or imaging study that has been shown to reliably assess remission of DFO. Both the IDSA and IWGDF guidelines [17–19] suggest combining various clinical findings (e.g., resolution of soft tissue inflammation and wound healing), laboratory markers (especially normalization of elevated CRP and ESR), and imaging tests (e.g., signs of radiological healing and absence of evidence of infection on advanced imaging) to determine the efficacy of treatment in DFO [93, 94]. However, the optimal combination and the accuracy and reproducibility of these tests in assessing the remission remain speculative. To address this clinical question, we found and retrieved 30 papers, only 6 of which met our criteria for inclusion. A prospective study evaluated 115 patients for the association between radiologic changes on serial X-rays and the development of long-term complications of diabetic foot OM at 1-year follow-up [95]. The authors reported that on follow-up, after proper treatment (with antibiotics and any required surgery), the presence of radiologic changes during the DFO clinical remission was significantly associated with the development of complications. Similarly, another recent retrospective review of 46 patients found that taking serial radiographs had an 80% diagnostic accuracy, with an 87% positive predictive value and 43% negative predictive value for diagnosing OM (*p* < 0.05) [96]. These results suggest that serial plain radiographs may be useful for both diagnosing OM and predicting complications.

MRI has been used in diabetic patients not only to diagnose foot OM but also to guide treatment decisions. In one retrospective study in patients with a neuropathic forefoot ulcer complicated by OM [55], sequential MRI scans were performed every 3 months during antibiotic therapy. Antibiotic therapy was continued unless the lesions healed and MRI findings improved. Using this treatment approach was associated with a high rate of healing without relapse, but a very (and likely unnecessarily) prolonged duration of antibiotic therapy (at least 6 months and up to several years). Another study in 32 patients with pathology-proven diabetic forefoot OM also demonstrated added value for MRI over X-rays alone in guiding surgical management in almost two-thirds of the patients, whether X-ray negative or positive [56].

Several studies have investigated the value of various NM procedures in managing diabetic foot OM. To investigate if SPECT/CT is useful to monitor treatment response in patients with DFO, Lazaga et al. retrospectively reviewed 20 patients who underwent ^99m^Tc-WBC SPECT/CT both at baseline and 7 months after initial therapy. They monitored for failure of wound healing or re-admission for the same ulcer within 1 year from the initial intervention, and found that SPECT/CT determined the remission with a PPV of 69% and NPV of 83% [97].

In 2014, Vouillarmet et al. aimed to assess the value of imaging tests in assessing remission of diabetic foot OM by performing WBC imaging, three-phase bone scan and X-rays in 29 patients at the end of their course of antibiotic treatment [98]. Sensitivity, specificity, PPV and NPV in predicting OM relapse at the end of therapy were, respectively, 100%, 91.5%, 71.5% and 100% for WBC scintigraphy + SPECT/CT; 100%, 12.5%, 15.5% and 100% for three-phase bone scan; and 80%, 33%, 20% and 89%, for X-rays. They concluded that a negative result on WBC imaging appeared to be a reliable tool for assessing post-treatment OM remission.

In 2017, the same group explored the feasibility of WBC SPECT/CT in predicting remission after 6 or 12 weeks of antibiotic treatment (without surgery) in 45 diabetic patients with foot OM. Scintigraphy was performed 6 weeks after initiating antibiotic therapy. If clinical signs and symptoms had not resolved or the scintigraphy was abnormal, treatment was continued for up to 12 weeks, when another WBC scintigraphy was performed. According to the results of WBC SPECT/CT, a 6 weeks course of antibiotic therapy was used in 23 patients, whereas in 22 patients treatment was extended to 12 weeks. After 6 weeks, all 23 patients had a negative WBC scan, but one patient experienced a relapse during follow-up, thus showing a NPV of 96%. In those treated for further 6 weeks, WBC scan at the end of antibiotic course was negative in 9 patients, all considered at remission (NPV: 100%) and was still positive in 13 patients (7 in remission and 6 in relapse). The authors concluded that this modality could be useful, not only to assess treatment response, but also to select patients who may benefit from more prolonged antibiotic therapy [99].

One paper not included in this PICO (since published in 1991), described the results of performing ^111^In oxyquinoline WBC scan every 2–3 weeks intervals during antibiotic treatment of diabetic foot OM. They reported a normalization of the scan results between 36 and 54 days after initiating treatment, and suggested that since this strategy may reliably assess for resolution of the OM it might be useful to perform it at least 4 weeks after the treatment has started [100].

Concluding, imaging might offer crucial information to help monitor the efficacy of therapy, especially if the same modality is performed at baseline and after therapy. There is, however, still a gap in literature on which is the most appropriate modality to employ and what is the optimal time of imaging during follow-up after an antibiotic therapy. A list of clinical questions and recommendations is shown in Table 1.

**Table 1:** Summary table of statements and recommendations

*N*	Clinical questions	References	Recommendations	Grade of recommendations
1	**a**: Is it true that patients with some combination of a positive PTB, changes in plain X-rays, local inflammatory signs, or elevated serological inflammatory markers (erythrocyte sedimentation rate, C-reactive protein, procalcitonin) have OM?	32, 36, 40–46, 48	A combination of a positive PTB test, an elevated ESR and a positive X-rays, makes the presence of OM highly probable and no additional imaging modalities are required for diagnosis.	B
	**b:** Do imaging modalities add any relevant information in patients with a combination of these clinical and laboratory findings used to diagnose OM?	15, 49, 50	Additional imaging with MRI may provide useful additional information on the extent of both bone and soft tissue infection.	
	**c:** What imaging modality should be performed in patients with positive results on one or more of these clinical and laboratory findings?	5, 7, 31, 32, 45, 50	If, however, PTB is the only positive test, advanced imaging modalities (MRI or WBC imaging or [^18^F]FDG PET/CT) should be considered, especially in high-risk patients.	
2	**a**: Which imaging modality is more accurate in diabetic patients with suspected OM of the fore-mid-foot?	7, 15, 31–36, 51–58	In case of equivocal results on MRI, radiolabelled WBC imaging or, in alternative, [^18^F]FDG PET/CT should be used to detect OM.	C
	**b**: Which modality is more accurate in cases of OM in the mid- or hind-foot?	15, 51, 57, 59–66		
3	**a:** What kind of imaging modality is recommended in patients with an ulcer located at fore-/ mid-foot complicated by a severe or moderate infection before performing a surgical debridement of the infected tissue?	15, 47, 70–77	MRI, WBC scan and [^18^F]FDG PET/CT have a comparable accuracy in detecting infected ulcers in any site. In case of equivocal MRI findings, hybrid imaging with both WBC scan and [^18^F]FDG PET/CT can be used.	C
	**b:** Which imaging modality is recommended if the infected ulcer is located in the hind-foot?	15, 51, 61, 70, 75		
4	Foot ulcers may complicate a CF, in both its acute and chronic stages and OM may develop. What imaging modality should be performed when there is suspicion of OM complicating a CF?	61, 64, 65, 70, 77–79, 81–84, 86, 87, 89–91	MRI has several limitations in diagnosing CF. WBC imaging is sensitive and specific for OM, even in presence of concomitant Charcot’s arthropathy. As an alternative, [^18^F]FDG PET/CT may be used.	B
5	What kind of examination should be performed to confirm that OM is cured after 6 weeks of antibiotic treatment?	55, 56, 95, 97–99	Advanced imaging (MRI and WBC scan) can provide useful information both during and after antibiotic therapy, to assess for resolution of infection and to decide if it is appropriate to stop or prolong therapy.	B

**Table:** 

***Recommendation***	***Grade***
Advanced imaging can provide useful information both during and after antibiotic therapy, to assess for resolution of infection and to decide if it is appropriate to stop or prolong therapy	*B*

## Future research agenda

From the analysis of current literature, several gaps emerged:

- Head-to-head comparisons between different imaging modalities for diagnosing foot complications are lacking. The optimal way to define the best diagnostic strategy, as has recently been done for vascular graft infections [101], would be comparing the accuracy of WBC scintigraphy, [^18^F]FDG PET/CT, and MRI in the same patient, using bone histology/microbiology as the gold standard.
- Many included studies were published before the “era” of hybrid imaging, thus possibly underestimating the diagnostic performance of both PET and WBC imaging. The use of stand-alone PET, planar scintigraphic images or SPECT acquisition without CT co-registration also represents a major limitation when comparing the accuracy of different imaging modalities.
- Standardization of acquisition protocols, as well as interpretation criteria, for all imaging modalities is warranted to reduce procedural heterogeneity and to allow optimal comparison amongst studies. This is particularly important for [^18^F]FDG PET/CT. Specifically, it would be very useful to identify the most accurate strategies for image interpretation to differentiate OM from STI and CF. This would allow different centres to achieve harmonization in their methods.
- It would be very useful to develop and attempt to validate and compare new specific protocols and sequences for MRI studies.
- Studies are required to better explore the role of late acquisitions (e.g., after 90–120 minutes from [^18^F]FDG injection) and dual-time-point PET/CT imaging for improving image quality and target/background ratios.
- Further studies are needed to investigate the possible role of PET/MRI in imaging diabetic foot infections.
- Further studies are needed to investigate the possible role of PET/MRI in imaging diabetic foot infections.
- Studies are needed to determine the results of application of more specific radiopharmaceuticals for infection imaging and therapy monitoring.
- Imaging is mostly employed for diagnosis, but is also important in the follow-up of patients with diabetic foot infections. Thus, data are needed from comparative longitudinal studies on the evolution of the infection. Particularly, investigating the possible impact of antibiotic therapy on NM imaging modalities would be helpful to better plan the diagnostic strategy.

Future research directed at filling these gaps would greatly increase the knowledge in this field and provide evidence that could be helpful in clinical practice for the management of specific clinical scenarios. Moreover, the availability, in each hospital, of a multi/inter-disciplinary team that jointly discuss difficult cases of diabetic foot complications has been shown to improve outcomes. Having specialists in radiology and NM play a role on these teams should help improve patient care [102].

## Conclusions and final recommendations

Clinical examination and blood tests are integral tools for clinicians assessing patients with suspected OM, but are often diagnostically insufficient. Thus, imaging studies play an important role in diagnosing, and sometime monitoring, diabetic foot complications, particularly the often-complex task of differentiating between OM, STI and Charcot arthropathy.

In agreement with current clinical guidelines published by IWGDF, we believe that in a diabetic patient with a foot wound and a positive PTB test, characteristic changes on plain X-rays and a substantially elevated ESR, the patient should be presumed to have OM, without the need for further imaging tests. In many patients, however, advanced imaging with MRI, WBC SPECT/CT, or [^18^F]FDG PET/CT may be needed. In particular, they should be considered when it is needed to better assess the location, extent or severity of an infection, in order to plan a more tailored treatment and to follow-up its efficacy. Although further studies are needed, it appears that MRI, radiolabelled WBC scan or perhaps [^18^F]FDG PET/CT could be used for monitoring therapy and determining if infection has resolved.

In a patient with a positive PTB test, normal or elevated ESR, but negative X-rays, OM cannot be excluded, given the long latency required for X-rays abnormalities to be seen. In these situations, advanced imaging with MRI or WBC scintigraphy + SPECT/CT, or as an alternative with [^18^F]FDG PET/CT, is often needed, either to make a more definitive diagnosis or to assess the results of treatment. In patients with a diabetic foot infection, microbiological (and in the case of suspected OM histological) assessment is the only way to determine the causative pathogens and their antibiotic susceptibilities. In a case with persisting clinical suspicion of OM but negative initial imaging studies, additional imaging should be considered.

In a case of suspected STI, MRI WBC scintigraphy and [^18^F]FDG PET/CT have a comparable accuracy and hybrid imaging is reserved in cases of un-conclusive MRI.

MRI is the modality of choice for the diagnosis of Charcot neuro-osteoarthropathy. However, if the clinical question is to assess if the CF has a superimposed infection, WBC SPECT/CT is the most effective technique to differentiate OM from Charcot arthropathy, possibly performed in combination with bone marrow scan. [^18^F]FDG PET/CT may be used as an alternative.

In most cases, decisions about ordering diagnostic imaging procedures are best made by multi/inter-disciplinary discussion with all professionals involved in the care of these patients. This approach allows tailored diagnostic and therapeutic strategies for each individual patient.

### Supplementary Information

Below is the link to the electronic supplementary material.

Supplementary file1 (DOCX 26 KB)

## Data Availability

Data are available upon request.

## Abbreviations

DFI: Diabetic foot infection
OM: Osteomyelitis
STI: Soft tissue infection
CF: Charcot’s foot
EANM: European Association of Nuclear Medicine
SPECT: Single-photon emission computed tomography
WBC: White blood cell
BS: Bone scan
MDP: Methylene di-phosphate
PET: Positron emission tomography
FDG: Fluorodeoxyglucose
SUV: Standardized uptake value
CT: Computed tomography
MRI: Magnetic resonance imaging
STIR: Short T1 inversion recovery
FAT-SAT: Fat saturation
OCEBM: Oxford Centre for Evidence-Based Medicine
PICO: Population/Problem–Intervention/Indicator–Comparator–Outcome
