# Supplementary material for: Diagnostic imaging of the diabetic foot: an EANM evidence-based guidance
Source: Eur J Nucl Med Mol Imaging. 2024 Mar 27;51(8):2229–46. doi: 10.1007/s00259-024-06693-y (PMC11178575; doi:10.1007/s00259-024-06693-y)
Supplement: Supplementary file 1 — Supplementary file1 (DOCX 26 KB) [file 259_2024_6693_MOESM1_ESM.docx]

# **APPENDIX**

**Supplementary table**

Description of search PICOs, search terms and paper found (and paper included for guidance) for each clinical question.

| **Question** | **Population Intervention Comparison Outcome** | | | | **Search terms** | **Papers found** | **Papers included** |
| --- | --- | --- | --- | --- | --- | --- | --- |
|  | **P** | **I** | **C** | **O** |  |  |  |
| **1** | diabetic patients with foot osteomyelitis | imaging modality | probe-to-bone test | diagnosis | (diabet* AND (foot AND (osteomyelitis OR infection)) AND (imaging OR MRI OR PET OR SPECT OR leukocyte* OR scintigraphy) AND (probe-to-bone test) AND (diagnosis)) | 23 | 17 |
| **2a** | diabetic patients with forefoot osteomyelitis | imaging modality | - | diagnosis | (diabet* AND (forefoot AND (osteomyelitis OR infection)) AND (MRI OR PET OR SPECT OR scintigraphy) AND (diagnosis)) | 28 | 16 |
| **2b** | diabetic patients with hindfoot osteomyelitis | imaging modality | - | diagnosis | (diabet* AND (forefoot AND (osteomyelitis OR infection)) AND (MRI OR PET OR SPECT OR scintigraphy) AND (diagnosis)) | 22 | 11 |
|  |  |  |  |  |  |  |  |
| **3a** | diabetic patients with forefoot soft tissue infection | imaging modality | - | diagnosis | (diabet*) AND (forefoot) AND (soft tissue) AND (infection OR ulcer) AND (imaging OR MRI OR PET OR SPECT OR scintigraphy) AND (diagnosis) | 25 | 11 |
| **3b** | diabetic patients with hindfoot soft tissue infection | imaging modality | - | diagnosis | (diabet*) AND (hindfoot) AND (soft tissue) AND (infection OR ulcer) AND (imaging OR MRI OR PET OR SPECT OR scintigraphy) AND (diagnosis) | 21 | 5 |
| **4** | diabetic patients with ulcer and Charcot | imaging modality | - | diagnosis of osteomyelitis | (diabet*) AND (Charcot) AND (osteomyelitis) AND (ulcer) AND (imaging OR MRI OR PET OR SPECT OR scintigraphy) AND (diagnosis) | 69 | 14 |
| **5** | diabetic patients with foot osteomyelitis | imaging modality | - | healing after antibiotic therapy | (diabet* AND (foot AND (osteomyelitis OR infection)) AND (imaging OR MRI OR PET OR SPECT OR scintigraphy) AND (healing)) | 454 | 6 |
